# Fragment molecular orbital-based variational quantum eigensolver for quantum chemistry in the age of quantum computing

**DOI:** 10.1038/s41598-024-52926-3

**Published:** 2024-01-29

**Authors:** Hocheol Lim, Doo Hyung Kang, Jeonghoon Kim, Aidan Pellow-Jarman, Shane McFarthing, Rowan Pellow-Jarman, Hyeon-Nae Jeon, Byungdu Oh, June-Koo Kevin Rhee, Kyoung Tai No

**Affiliations:** 1 Bioinformatics and Molecular Design Research Center (BMDRC), Incheon, Republic of Korea; 2 QuNova Computing, Inc., Daejeon, Republic of Korea; 3 Baobab AiBIO Co., Ltd., Incheon, Republic of Korea; 4 SKKU Advanced Institute of Nanotechnology, Sungkyunkwan University, Suwon, Republic of Korea

**Keywords:** Theoretical chemistry, Quantum chemistry

## Abstract

Quantum computers offer significant potential for complex system analysis, yet their application in large systems is hindered by limitations such as qubit availability and quantum hardware noise. While the variational quantum eigensolver (VQE) was proposed to address these issues, its scalability remains limited. Many efforts, including new ansätze and Hamiltonian modifications, have been made to overcome these challenges. In this work, we introduced the novel Fragment Molecular Orbital/Variational Quantum Eigensolver (FMO/VQE) algorithm. This method combines the fragment molecular orbital (FMO) approach with VQE and efficiently utilizes qubits for quantum chemistry simulations. Employing the UCCSD ansatz, the FMO/VQE achieved an absolute error of just 0.053 mHa with 8 qubits in a ${{\text{H}}}_{24}$ system using the STO-3G basis set, and an error of 1.376 mHa with 16 qubits in a ${{\text{H}}}_{20}$ system with the 6-31G basis set. These results indicated a significant advancement in scalability over conventional VQE, maintaining accuracy with fewer qubits. Therefore, our FMO/VQE method exemplifies how integrating fragment-based quantum chemistry with quantum algorithms can enhance scalability, facilitating more complex molecular simulations and aligning with quantum computing advancements.

## Introduction

Quantum mechanical (QM) methods have been utilized to address a wide range of chemical problems, providing precise descriptions of structural interactions within targeted systems. However, these methods come with a rapidly escalating computational cost, with even the most basic Hartree–Fock (HF) method scaling at least as $O({N}^{3-4})$ with the system size *N*^1^. Highly accurate calculations, such as those involving coupled-cluster (CC) methods, can be performed to attain chemical accuracy within 1 kcal/mol, given the use of a large basis set for small molecules. As the number of electrons and basis functions increases, the use of CC methods with single and double excitations (CCSD) and triple excitations (CCSD(T)) becomes progressively more time-intensive, with the computational cost scaling as $O({N}^{6})$ for CCSD and $O({N}^{7})$ for CCSD(T)^2^. To reduce these escalating costs while maintaining accuracy, many computational methods have been developed to improve the efficiency and scalability of quantum chemistry simulations^2^.

As an effective strategy to balance the conflicting demands of accuracy and speed, fragment-based quantum chemistry methods were proposed. These methods operate by dividing a large system into more manageable smaller fragments on which electronic structure calculations are more feasible. The primary advantage of this divide-and-conquer strategy lies in its capacity to substantially enhance computational efficiency without severely compromising accuracy. For example, the fragment molecular orbital (FMO) method was developed by Kitaura et al. in 1999 as a practical way to apply energy decomposition analysis to larger systems^3^. The FMO method promotes more efficient parallel processing by individually addressing smaller fragments, thereby enabling the simulation of larger systems with a reasonable computational cost. Moreover, the FMO method considers the electrostatic potential of the entire system in individual fragment calculations, making it retain its accuracy when compared to traditional QM methods. It has been successfully employed in a range of applications, including the analysis of materials such as zeolites, nanowires, and nanoparticles, and biological systems with small ligands, DNA, RNA, and proteins^4–11^. Despite its enhanced computational efficiency and successful applications, the FMO method still has high computational costs when applied to highly accurate analysis on larger systems with the use of the CC method and large basis sets. Therefore, there remains a critical need for additional strategies to further reduce computational costs.

Quantum computers, leveraging unique quantum properties such as superposition, interference, and entanglement, are expected to significantly reduce the computational burden associated with quantum chemistry calculations. Such a shift could revolutionize the field of quantum chemistry by enabling the calculation of the ground state energy of molecular Hamiltonians and solving complex electronic structure problems. Central to this quantum acceleration, the quantum phase estimation (QPE) algorithm can exponentially determine the eigenstates and eigenvalues of unitary operators, including the electronic Hamiltonian, provided a suitable trial state is prepared^12–14^. However, the full implementation of QPE necessitates a considerable number of qubits, a demand currently strained by existing technological limitations. Moreover, as discussed in Lee et al.^15^, realizing an immediate exponential quantum advantage in applications such as ground-state energy estimation remains elusive due to the complexities involved in quantum state preparation. Given the early stages of quantum computers, there is a growing interest in practical applications for devices with limited capabilities, known as noisy intermediate-scale quantum (NISQ) devices. These NISQ devices, which operate with fewer qubits, inherently show significant error rates. Therefore, error mitigation algorithms have become essential for managing these errors and additionally, hybrid algorithms have frequently been employed to increase operational efficiency by leveraging both quantum and classical computations.

The variational quantum eigensolver (VQE) is a hybrid algorithm that combines classical and quantum processing to solve eigenvalue problems, making it aptly suited for the NISQ devices currently available. Introduced initially by Peruzzo et al.^16^ and later refined by McClean et al.^17^ and Romero et al.^18^, the VQE primarily aims to find an upper limit for the ground state energy of a Hamiltonian in quantum chemistry. This involves constructing a ground state trial wave function using the HF method and the unitary ansatz, often the UCCSD ansatz, on a classical computer, then preparing and measuring it on a quantum computer, followed by the optimization of its parameters using the variational principle^19–25^. The VQE has been applied to studying the potential energy surfaces of small molecules and bimolecular nucleophilic substitution reactions^16,26–28^. Nevertheless, the scalability of the VQE is inherently constrained not only by the finite number of qubits in NISQ devices, which restricts its applicability but also by the complexities of both the quantum circuits and the classical optimization problems. Numerous efforts have been made to address these limitations while maintaining high accuracy, such as reducing the depth of quantum circuits, simplifying the electronic Hamiltonian, and reducing measurement overhead^26,29–31^. Ansätze such as k-UpCCGSD^32^, ADAPT-VQE^33^, DUCC^34^, OO-UCC^35^, qubit-ADAPT-VQE^36^, and SPA^37^ have been developed to minimize quantum circuit size. The Separable Pair Ansatz (SPA) was also developed to combine the hard-core boson model and Jordan–Wigner to produce shallow, memory-efficient quantum circuits^37^. The resulting wave function has a product structure of individual pair functions that are coupled through the Hamiltonian, essentially defining a mean-field model for pairs^37^. Additionally, to reduce the electronic Hamiltonian, quantum embedding theories such as dynamical mean field theory^38^ and density matrix embedding theory^39^ have been integrated with the VQE^26^. These methods were designed to algorithmically complement the limited qubit capacity of NISQ devices, thereby broadening their scope, even in investigating protein–ligand interactions for drug design^40^. Concerning the measurement overhead required by VQE, the number of shots required to estimate the energy of a Hamiltonian of *N* Pauli words to some accuracy $\varepsilon$ in the base case is $O\left(\frac{{N}^{4}}{{\varepsilon }^{2}}\right)$ ^26^; however, methods have been developed to reduce this, such as using the commutativity of the Pauli words in the Hamiltonian to group terms for simultaneous measurement^26^.

In this study, we introduced a novel quantum computing algorithm, FMO/VQE, which combines the fragment molecular orbital method with the variational quantum eigensolver. We verified the accuracy of the FMO/VQE algorithm by comparing the ground-state energies of hydrogen clusters obtained using classical FMO and QM methods. We first optimized the structures of cationic, neutral, and anionic hydrogen clusters using traditional QM methods. Next, we employed our UCCSD and QCC ansätze and validated their performance on the systems. We then implemented these ansätze within the FMO/VQE algorithm and validated them in neutral hydrogen clusters ranging from ${{\text{H}}}_{6}$ to ${{\text{H}}}_{24}$. Finally, we extended the application of FMO/VQE to anionic hydrogen clusters from ${{\text{H}}}_{3}^{-}$ to ${{\text{H}}}_{23}^{-}$. We found that the FMO/VQE approach can significantly reduce the computational resources required for quantum simulations of complex molecular systems, paving the way for the application of quantum computing to a wide range of chemical and materials science problems.

## Methods

### The electronic structure Hamiltonian

The electronic structure problem refers to the task of finding the lowest energy levels of chemical systems. The ab initio molecular Hamiltonian is an operator for the total energy of any molecular system based on its atomic coordinates, which include atomic compositions and relative positions of the nuclei. To obtain the electronic wave function at the ground state energy level, it is necessary to determine the correlated probability amplitudes of the electrons within the space around the nuclei, depending on the molecular system. In a non-relativistic setting, it is assumed that the heavier nuclei motion on a much slower time scale compared to electrons and their behaviors can be treated as decoupled from each other, based on the Born–Oppenheimer approximation^1^. The Hartree–Fock (HF) method approximates the N-body wave function of a molecular system by a single N-body spin determinant orbital, in which each electron is assumed to evolve in the mean-field created by all other electrons in the system. Through the self-consistent field (SCF) theory^1^, the mean-field created by the other electrons is determined self-consistently, meaning that it is iteratively updated until convergence is achieved. The resulting single determinant wavefunction is used to calculate the electronic energy of the system, but the HF method has its limitations, particularly for strongly correlated systems^1^.

### Fragment molecular orbital (FMO) calculations

Fragment molecular orbital (FMO) is a computational method for approximating the electronic structure of large molecules by dividing them into smaller fragments and then calculating the electronic structure of each fragment separately. The two-body FMO-based restricted Hartree–Fock (FMO-RHF) calculation involves four steps: fragmentation, monomer SCF calculation, dimer SCF calculation, and total property evaluation. Firstly, the whole system is divided into individual fragments. Each hydrogen molecule or ion in hydrogen clusters can be defined as a fragment. Secondly, the MOs on each fragment (monomer) are optimized by the SCF theory in the external electrostatic potential generated by the surrounding *N* − 1 fragments, with all-electron densities solved through self-consistent-charge iterations^3,7,41^. The Hamiltonian for the monomer is given by,

1 $$\hat{H}_{I} \Psi_{I} = E_{I} \Psi_{I} ,$$

2 $$\hat{H}_{I} = \mathop \sum \limits_{i \in I} \left[ { - \frac{1}{2}\Delta_{i} - \mathop \sum \limits_{A} \frac{{Z_{A} }}{{\left| {r_{i} - R_{A} } \right|}} + \mathop \sum \limits_{J \ne I}^{{N_{f} }} \smallint dr^{\prime}\frac{{\rho_{J} \left( {r^{\prime}} \right)}}{{\left| {r_{i} - r^{\prime}} \right|}}} \right] + \mathop \sum \limits_{i \in I} \mathop \sum \limits_{i < j \in I} \frac{1}{{\left| {r_{i} - r_{j} } \right|}},$$

where the entities $I$ and $J$ represent different fragments and ${\rho }_{J}\left({r}{\prime}\right)$ denotes the electron density with respect to the coordinate ${r}{\prime}$ in the fragment $J$. Just as in traditional QM where the wavefunction and energy are derived by solving the Schrödinger equation, the same approach is followed in FMO to obtain the wavefunction and energy for each fragment. Thirdly, the MOs of a fragment pair (dimer) are solved self-consistently in the same way as for the monomer calculations. However, the Hamiltonian operator used for the dimer calculation includes the external electrostatic potential generated by the surrounding *N* − 2 fragments and is given by,

3 $$\hat{H}_{IJ} \Psi_{IJ} = E_{IJ} \Psi_{IJ} ,$$

4 $$\hat{H}_{IJ} = \mathop \sum \limits_{i \in I, J} \left[ { - \frac{1}{2}\Delta_{i} - \mathop \sum \limits_{A} \frac{{Z_{A} }}{{\left| {r_{i} - R_{A} } \right|}} + \mathop \sum \limits_{K \ne I,J}^{{N_{f} }} \smallint dr^{\prime}\frac{{\rho_{J} \left( {r^{\prime}} \right)}}{{\left| {r_{i} - r^{\prime}} \right|}}} \right] + \mathop \sum \limits_{i \in I,J} \mathop \sum \limits_{i < j \in I,J} \frac{1}{{\left| {r_{i} - r_{j} } \right|}},$$

where the entity $K$ refers to a different fragment. The fourth step involves piecing together all of the MO results for the monomer and dimer fragments to generate a comprehensive description of the system and to determine overall properties such as energy and gradient,

5 $$E^{{{\text{FMO}}2 - {\text{RHF}}}} = E^{{{\text{FMO}}1 - {\text{RHF}}}} + \Delta E^{{{\text{FMO}}2 - {\text{RHF}}}} ,$$

6 $$E^{{{\text{FMO}}1 - {\text{RHF}}}} = \mathop \sum \limits_{I}^{N} E_{I} ,$$

7 $$\Delta E^{{{\text{FMO}}2 - {\text{RHF}}}} = \mathop \sum \limits_{I}^{N} \left[ {\left( {E_{IJ} - E_{I} - E_{J} } \right)} \right],$$

where $N$ is the number of fragments in the system and ${E}_{I}$ and ${E}_{IJ}$ are the electrostatically embedded energies in the monomer and dimer, respectively.

Coupled-cluster (CC) methods build upon the HF method by adding the electron correlation energy. They provide a more accurate representation of molecular properties and are generally accepted as a good standard for accurate QM calculations. The FMO-CC method, which is an FMO-based single-reference CC method, was developed to have linear computational scaling and can be parallelized, which involves adding the electron correlation energy to the FMO-RHF energy^42^,

8 $$E^{{{\text{FMO}}n - {\text{CC}}}} = E^{{{\text{FMO}}n - {\text{RHF}}}} + E^{{{\text{FMO}}n - {\text{corr}}}} ,$$

9 $$E^{{{\text{FMO}}2 - {\text{corr}}}} = E^{{{\text{FMO}}1 - {\text{corr}}}} + \Delta E^{{{\text{FMO}}2 - {\text{corr}}}} ,$$

10 $$E^{{{\text{FMO}}1 - {\text{corr}}}} = \mathop \sum \limits_{I}^{N} E_{I}^{{{\text{corr}}}} ,$$

11 $$\Delta E^{{{\text{FMO}}2 - {\text{corr}}}} = \mathop \sum \limits_{I > J}^{N} \left( {E_{IJ}^{{{\text{corr}}}} - E_{J}^{{{\text{corr}}}} - E_{I}^{{{\text{corr}}}} } \right),$$

where $N$ is the number of fragments in the system and ${E}_{I}^{{\text{corr}}}$ and ${E}_{IJ}^{{\text{corr}}}$ are the CC correlation energies in the monomer and dimer, respectively. The FMO-CC computational scheme is akin to that of FMO-RHF, but with CC calculations being performed on monomers after the monomer SCF calculation converges and on dimers after the dimer calculation converges. The FMO-CC calculations only consider occupied and virtual orbitals, and chemical core orbitals are not included in the computation. The two-body FMO methods inherently exhibit nearly linear $O(N)$ scaling, a characteristic consistently maintained across n-body expansions and applicable to both correlated and uncorrelated wave functions^41–43^. The classical FMO calculations were performed in GAMESS^5^ with the version Sep 30, 2022.

### Jordan–Wigner mapping

By using the canonical fermionic anti-commutation introduced by Jordan and Wigner^44^, the electronic Hamiltonian can be reconstructed through a straightforward projection onto the single-particle basis function. The conversion of the electronic structure problem into quantum states that can be processed by quantum computers necessitates the transformation of fermionic operators in the Hamiltonian into spin operators, which can be performed through the mapping methods. The Jordan–Wigner (JW) mapper allows qubits to directly represent the occupation of a given spin-orbital and shows the qubit operations of order $O\left(n\right)$. The JW mapping uses one qubit per spin-orbital and encodes the occupancy of the orbital in the state of the qubit. A detailed explanation of the JW mapping can be found in some papers^26,45,46^.

### Variational quantum eigensolver (VQE) calculations

Variational quantum eigensolver (VQE) was first introduced by Peruzzo et al.^16^ and extended by McClean et al.^17^. The VQE is developed to compute the ground state energy of a Hamiltonian and is based on the variational principle, which optimizes an upper bound for the lowest possible expectation value of an observable given a trial wavefunction^26^. The primary objective of the VQE is to minimize the expectation value of the Hamiltonian for a given trial wave function by finding the optimal set of parameters. This expectation value represents an upper limit on the ground state energy and, ideally, should be the same as the actual ground state energy within the desired level of accuracy. To implement this minimization task on a quantum computer, the trial wavefunction can be prepared using a parameterized quantum circuit known as an ansatz.

An ansatz generates the trial state that is used to measure the Hamiltonian and successful optimization of the ansatz parameters can produce a model for the ground state wavefunction. The choice of the parameterized ansätze greatly affects the performance of the VQE and much effort has been invested into designing accurate and efficient ansätze. Two essential aspects of the ansätze are its expressibility and trainability. The expressibility determines how well it can approximate the relevant low-energy states. It relies on having a good initial approximation to the eigenstate of the Hamiltonian since their performance is highly sensitive to the quality of the initial guess provided. The trainability relates to its practical optimization on quantum devices. The scaling and complexity of the ansatz circuit depth with system size are also important considerations, particularly for near-term VQE applications, as they can impact the noise resilience of the method.

A unitary coupled-cluster (UCC) is a physically inspired framework that can approximate the exact eigenstates of the Hamiltonian by increasing the rank of the excitation operators^32^. The UCC approach constructs a trial state by considering excitations beyond the initial reference state and can converge even when using multireference initial states, which is not always possible with the CC method. The UCC ansatz is typically truncated at a certain excitation level, commonly including single and double excitations, and this reduced form is referred to as UCCSD and can be expressed as,

12 $$\left| {\Psi_{{{\text{UCCSD}}}} } \right\rangle = e^{{\hat{T} - \hat{T}^{\dag } }} \left| {\Psi_{{{\text{RHF}}}} } \right\rangle ,$$

13 $$\hat{T} = \hat{T}_{1} + \hat{T}_{2} ,$$

14 $$\hat{T}_{1} = \mathop \sum \limits_{i, a} t_{i}^{a} \hat{a}_{a}^{\dag } \hat{a}_{i} ,$$

15 $$\hat{T}_{2} = \frac{1}{4}\mathop \sum \limits_{i,j, a,b} t_{ij}^{ab} \hat{a}_{a}^{\dag } \hat{a}_{b}^{\dag } \hat{a}_{j} \hat{a}_{i} ,$$

where ${\widehat{T}}_{1}$ and ${\widehat{T}}_{2}$ are the single and double excitations, the indices $i$, $j$ are occupied orbitals, and the indices $a$, $b$ are unoccupied orbitals. The UCC is widely used due to its robustness and accuracy^22^, but it can cause a growth in the number of simultaneously entangled qubits, which not all quantum computing architectures can handle. On the other hand, qubit coupled-cluster (QCC) was proposed as an alternative to UCC, which can be hindered by non-local action arising from the significant number of two-qubit gates required^47^. The QCC employs a different approach by using spin operators directly to construct the ansatz in the qubit space, avoiding the use of fermionic excitation operators that would require transformation. The QCC eliminates the need for fermion-to-qubit mappings, so it can reduce the computational cost. Moreover, the QCC with the factorization technique uses only two-qubit entanglement gates, allowing for efficient use of quantum resources. The QCC wave function can be expressed as follows:

16 $$\left| {\psi \left( {\tau , {\Omega }} \right)} \right\rangle = \hat{U}\left( \tau \right)\left| {\Omega } \right\rangle ,$$

17 $$\left| {\Omega } \right\rangle = \mathop \prod \limits_{i = 1}^{{N_{q} }} \left| {{\Omega }_{i} } \right\rangle ,$$

18 $$\left| {{\Omega }_{i} } \right\rangle = \cos \left( {\frac{{\theta_{i} }}{2}} \right)\left| {\upalpha } \right\rangle + e^{{i\phi_{i} }} \sin \left( {\frac{{\theta_{i} }}{2}} \right)\left| {\upbeta } \right\rangle ,$$

where $\phi_{i}$ and $\theta_{i}$ are the azimuthal and polar angles of the $i{\text{th}}$ qubit, respectively, while $\left| {\upalpha } \right\rangle$ and $\left| {\upbeta } \right\rangle$ are spin-up and spin-down eigenstates of the $\hat{s}_{z} \left( i \right) = \frac{1}{2}\hat{z}_{i}$ operator.

As mentioned in “Introduction” section, there is a variety of different ansätze that have been developed for VQE with varying motivations and advantages, however in this study, we limit the scope of our investigation to the UCCSD and QCC ansätze to showcase the FMO/VQE method with a standardized VQE approach.

### Fragment molecular orbital-based variational quantum eigensolver (FMO/VQE)

To run the VQE on monomer or dimer fragments generated by OpenFMO^48^ for the FMO/VQE, the system information in each fragment needs to be provided to the VQE. The information is a collection of critical data including one- and two-electron integrals, the nuclear repulsion energy, molecular orbitals’ occupation numbers and RHF energies, the number of electrons within the system, the active space’s orbital lists, and the orbitals designated to be frozen. The OpenFMO is utilized to produce this comprehensive set of system information. The active space is chosen by specifying how many orbitals to select around the highest occupied molecular orbital and the lowest unoccupied molecular orbital. The relevant VQE settings, such as which ansatz and optimizer to use, are specified in the call to the VQE. This information is all provided to the VQE through a secure server connection and is then used to initialize and run the VQE algorithm. The one-electron integrals, two-electron integrals, and nuclear repulsion energy are all used to build the fermionic Hamiltonian. If the active space is not provided, the full Hamiltonian is generated. If provided, only the integrals for the orbitals in the active space are included, thereby defining the active Hamiltonian. This Hamiltonian is transformed into a Pauli Hamiltonian using the specified mapping. Though the orbital energies and occupation numbers aren’t necessary when using the QCC ansatz, if the UCCSD ansatz is selected then this information is used in the calculation of initial amplitudes for the excitations. These initial amplitudes allow the estimated contribution of each excitation to be considered and then screened using a specified threshold, reducing the computational cost of these ansätze through a reduction in terms. Once the VQE has finished running, the resulting energies are sent back to OpenFMO for further processing. Here, we used the full Hamiltonian without active space, the JW transformation, the quasi-Newton optimizer (SLSQP)^49^, and the threshold of ${10}^{-3}$ for initial amplitudes and considered all entanglements in the specified ansätze (QCC and UCCSD). Each hydrogen molecule, cation, and anion is defined as a fragment. In the FMO/VQE, monomer and dimer calculations require a maximum of 4 and 8 qubits, respectively, when using the STO-3G basis set. Moreover, these calculations demand up to 8 and 16 qubits when applying the 6-31G basis set.

### Structure preparation

Three sets of test molecules were composed and prepared. The purpose of the first set is to validate two VQE algorithms (QCC and UCCSD ansätze) by comparing the results to those from ab initio CCSD (QM/CCSD). Due to the limited qubit numbers of the state-vector simulator, three cationic ${{\text{H}}}_{{\text{n}}}^{+}$ clusters ($n=3$, $5$, $9$) and the two basis sets (STO-3G and 6-31G) were used. The second test set was constructed to validate the FMO/VQE algorithms by comparing the results to those from QM/CCSD and FMO/CCSD, where ten neutral ${{\text{H}}}_{{\text{n}}}$ clusters ($n = 6$, $8$, $\ldots$, $24$) and two basis sets (STO-3G and 6-31G) were used. The purpose of the third test set was to apply the FMO/VQE algorithms to analyze the anionic hydrogen clusters, where nine ${{\text{H}}}_{{\text{n}}}^{-}$ clusters ($n = 5$, $7$, $\ldots$, $21$) and the 6-31G basis set were used. The geometries of the cationic and neutral hydrogen clusters were obtained by full optimization at the aug-cc-pVDZ/CCSD level, while the structures of anionic hydrogen clusters were derived from Calvo et al.^50^. All quantum chemistry calculations of QM/CCSD and FMO/CCSD were performed in GAMESS^5^ with the version Sep 30, 2022, while all calculations of FMO/VQE were performed with OpenFMO^48^.

## Results

**Figure 1:**
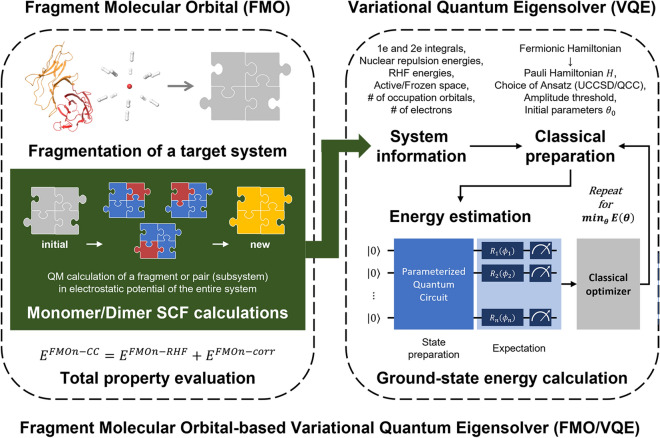
The workflow of fragment molecular orbital-based variational quantum eigensolver (FMO/VQE).

The fragment molecular orbital-based variational quantum eigensolver (FMO/VQE) approach is grounded on the FMO framework and its process is illustrated in Fig. 1. By integrating independent SCF calculations on FMO with the VQE, the FMO/VQE framework reduces the number of required qubits for the same system compared to the VQE, thereby providing a more efficient alternative. The traditional VQE approaches often require a substantial number of qubits, normally twice the number of molecular orbitals, for accurately estimating the system’s energy. However, the FMO/VQE alleviates this burden by employing a strategy that performs the SCF calculations on individual fragments and leverages the VQE to estimate the electron correlation energy of the fragments, effectively lowering the overall qubit requirements. In this framework, each SCF calculation is coupled with a VQE call, substituting conventional post-Hartree–Fock methods, such as the CCSD, with a quantum computational algorithm. After all SCF calculations of all monomers and dimers converge, the individually obtained fragment energies, inclusive of the electron correlation contributions from the VQE, are then amalgamated to estimate the total system energy. To validate the efficiency and accuracy of the FMO/VQE as a highly effective, practical, and reliable alternative in comparison to traditional VQE methods, we applied the FMO/VQE to hydrogen cluster systems.

To validate the VQE ansatz, we prepared a range of hydrogen cluster systems with positive, neutral, and negative charge states and summarized the VQE results in Table 1. These systems include different charge states of hydrogen atoms (cation, neutral, and anion): three cationic systems (${{\text{H}}}_{3}^{+}$, ${{\text{H}}}_{5}^{+}$, and ${{\text{H}}}_{9}^{+}$), three neutral systems (${{\text{H}}}_{6}$, ${{\text{H}}}_{8}$, and ${{\text{H}}}_{10}$), and four anionic systems (${{\text{H}}}_{3}^{-}$, ${{\text{H}}}_{5}^{-}$, ${{\text{H}}}_{7}^{-}$, and ${{\text{H}}}_{9}^{-}$). Each structure of these systems was used for quantum chemistry simulations in a state-vector simulator with varying numbers of qubits depending upon system sizes and basis sets (STO-3G and 6-31G). However, the application of the VQE ansatz to larger systems was not feasible because the number of qubits required by the calculations surpassed the maximum capacity of 32 qubits offered by the state-vector simulator.

**Table 1:** The error of VQE relative to QM/FCI in hydrogen cluster systems.

Class	System	Basis set	Qubit	Absolute Energy Error (mHa)			Relative accuracy (%)		
				QM/CCSD	VQE/QCC	VQE/UCCSD	QM/CCSD	VQE/QCC	VQE/UCCSD
Cation	H_3_^+^	STO-3G	6	0.000	0.181	0.000	99.99999	99.98570	100.00000
	H_5_^+^		10	0.160	0.849	0.164	99.99339	99.96486	99.99322
	H_9_^+^		18	0.128	2.391	0.195	99.99726	99.94900	99.99583
	H_3_^+^	6-31G	12	0.000	0.239	0.000	100.00000	99.98167	99.99996
	H_5_^+^		20	0.356	1.643	0.174	99.98545	99.91841	99.97835
Neutral	H_6_	STO-3G	12	0.001	1.781	0.001	99.99997	99.94777	99.99996
	H_8_		16	0.002	3.558	0.054	99.99995	99.92169	99.99882
	H_10_		20	0.003	7.236	0.136	99.99995	99.87268	99.99761
Anion	H_3_^−^	STO-3G	6	0.000	0.015	0.001	100.00000	99.99888	99.99991
	H_5_^−^		10	0.000	0.124	0.001	100.00000	99.99490	99.99998
	H_7_^−^		14	0.000	1.707	0.023	100.00000	99.95231	99.99936
	H_9_^−^		18	0.000	3.358	0.016	100.00000	99.92887	99.99966
	H_3_^−^	6-31G	12	0.034	0.682	0.134	99.99788	99.95718	99.99160
	H_5_^−^		20	0.082	2.344	0.293	99.99702	99.91493	99.98937

To evaluate the accuracy of the VQE ansatz, we compared the performance of QM/CCSD, VQE/QCC, and VQE/UCCSD algorithms against the ab initio values at the full configuration interaction level (QM/FCI). The QM/FCI method provides an exact solution to the Schrödinger equation for a specific basis set and considers all possible excitations of electrons, so the QM/FCI serves as the gold standard in quantum chemistry for a given basis set. The discrepancies in absolute energy in milliHartree (mHa) and relative accuracy (%) were measured between our calculated results and the QM/FCI values. The accuracy of quantum calculations, often termed chemical accuracy, is typically set to a threshold of approximately 1.594 mHa, equivalent to 1 kcal/mol. This gives us an acceptable margin of error for these complex calculations and ensures the effectiveness and reliability of quantum chemistry methods.

To establish a comparative baseline for the VQE and FMO/VQE algorithm evaluation, we compared the results from the well-established QM/CCSD and QM/FCI methodologies. The QM/CCSD method is a highly accurate ab initio method and has shown chemical accuracy compared to QM/FCI. With its robust performance across hydrogen cluster systems, the QM/CCSD showed minimal absolute energy errors and consistently high relative accuracy. Specifically, the QM/CCSD showed its biggest error in ${{\text{H}}}_{5}^{+}$ of 0.160 mHa in STO-3G and 0.356 mHa in 6-31G basis set and achieved very low absolute energy errors of 0.055 mHa across all the systems on average. Likewise, in relative accuracy, the QM/CCSD showed its lowest accuracy in ${{\text{H}}}_{5}^{+}$ of 99.99339% in STO-3G and 99.98545% in the 6-31G basis set and achieved a very high relative accuracy of 99.99792% across all the systems on average. Therefore, the QM/CCSD achieved very similar accuracy to the QM/FCI and showed chemical accuracy in all hydrogen clusters with substantially reduced computational costs, compared to the QM/FCI.

Next, we evaluated the performance of the VQE/QCC and VQE/UCCSD ansätze against the gold standard QM/FCI. Our initial focus was the simplest system of ${{\text{H}}}_{3}^{+}$, employing the simplest STO-3G basis set. Both the VQE/QCC and VQE/UCCSD demonstrated good performance with very low absolute energy error (0.181 mHa and 0.000 mHa, respectively) and high relative accuracy (99.98570% and 100%, respectively), suggesting a proficient ground for each method in managing systems of lower complexity. However, the performance gap between the two ansätze became more pronounced as they were applied to increasingly larger systems, up to ${{\text{H}}}_{9}^{+}$. The VQE/QCC demonstrated a rising trend in absolute energy error, while VQE/UCCSD maintained its laudable consistency with minimal energy error increases. This pattern persisted even when the basis set was changed to the more complex 6-31G. Despite the greater qubit requirements due to larger systems or higher basis sets, VQE/UCCSD managed to match the performance of QM/FCI in terms of low absolute energy errors, but the VQE/QCC consistently showed relatively high absolute energy errors.

When examining neutral and anionic systems, a similar trend was observed. The VQE/UCCSD maintained commendable performance metrics, delivering low energy errors and high relative accuracies, even with increasing system complexity. On the other hand, the performance of the VQE/QCC was less consistent, exhibiting larger energy errors and achieving the highest absolute energy error of 7.236 mHa in the biggest system (${{\text{H}}}_{10}$). One particularly noteworthy observation is the consistency in the performance of the VQE/QCC and VQE/UCCSD across hydrogen cluster systems with different charge states. This robust behavior underscores the adaptability of these quantum computational methods in handling a diverse range of charged systems. Therefore, the VQE/UCCSD showed remarkable resilience and effectiveness against system size expansion. Even when higher numbers of qubits were required, the VQE/UCCSD maintained its accuracy within the chemical accuracy threshold of 1.594 mHa. It emphasizes the inherent strength of the VQE/UCCSD method in managing more complex quantum systems without compromising on the accuracy of energy calculations.

After we validated the performance of the VQE ansatz, we integrated the VQE ansatz into an FMO framework to make the FMO/VQE. To validate the performance of the FMO/VQE, we performed the FMO/VQE calculations with the neutral hydrogen cluster systems. These systems range from the cluster of three hydrogen molecules, denoted as ${{\text{H}}}_{6}$, to the cluster consisting of twelve hydrogen molecules, represented as ${{\text{H}}}_{24}$. The calculations employed two classical references (QM/CCSD and FMO/CCSD) and two quantum computational ansätze (FMO/VQE/QCC and FMO/VQE/UCCSD) with two different basis sets (STO-3G and 6-31G). The results from the FMO/VQE on the neutral clusters are summarized in Table 2, where the absolute error and relative accuracies were measured against the ab initio values derived from the QM/CCSD. Although the QM/FCI is widely accepted as the gold standard in quantum chemistry, its extensive computational requirements often limit its application to smaller systems. The QM/CCSD, conversely, achieves similar accuracy to QM/FCI but with substantially reduced computational costs. Thus, by setting QM/CCSD as our reference standard, we can establish a valid benchmark for accuracy and enable a more efficient validation of our FMO/VQE algorithm.

**Table 2:** The error of FMO/VQE relative to QM/CCSD in neutral systems.

System	Basis set	Absolute energy error (mHa)			Relative accuracy (%)		
		FMO/CCSD	FMO/VQE/QCC	FMO/VQE/UCCSD	FMO/CCSD	FMO/VQE/QCC	FMO/VQE/UCCSD
H_6_	STO-3G	3.863	0.332	0.007	99.88673	99.99025	99.99980
H_8_		7.171	1.132	0.016	99.84227	99.97511	99.99965
H_10_		10.267	1.286	0.016	99.81935	99.97386	99.99972
H_12_		15.449	1.378	0.011	99.77346	99.97979	99.99984
H_14_		22.831	4.197	0.055	99.71303	99.94724	99.99930
H_16_		30.035	3.141	0.107	99.66968	99.96545	99.99883
H_18_		45.098	5.280	0.076	99.55913	99.94838	99.99926
H_20_		50.275	4.572	0.082	99.55768	99.95977	99.99927
H_22_		51.012	4.499	0.142	99.59198	99.96402	99.99886
H_24_		48.148	3.216	0.053	99.64700	99.97642	99.99961
H_6_	6-31G	0.211	3.895	0.026	99.99390	99.88725	99.99925
H_8_		0.668	6.248	0.316	99.98550	99.86434	99.99314
H_10_		0.481	11.101	0.432	99.99164	99.80718	99.99250
H_12_		0.753	12.251	0.581	99.98910	99.82267	99.99158
H_14_		1.260	21.076	1.053	99.98437	99.73850	99.98693
H_16_		0.078	22.535	1.456	99.99915	99.75535	99.98420
H_18_		0.057	30.553	1.588	99.99945	99.70516	99.98467
H_20_		0.051	32.331	1.376	99.99956	99.71922	99.98805

**Figure 2:**
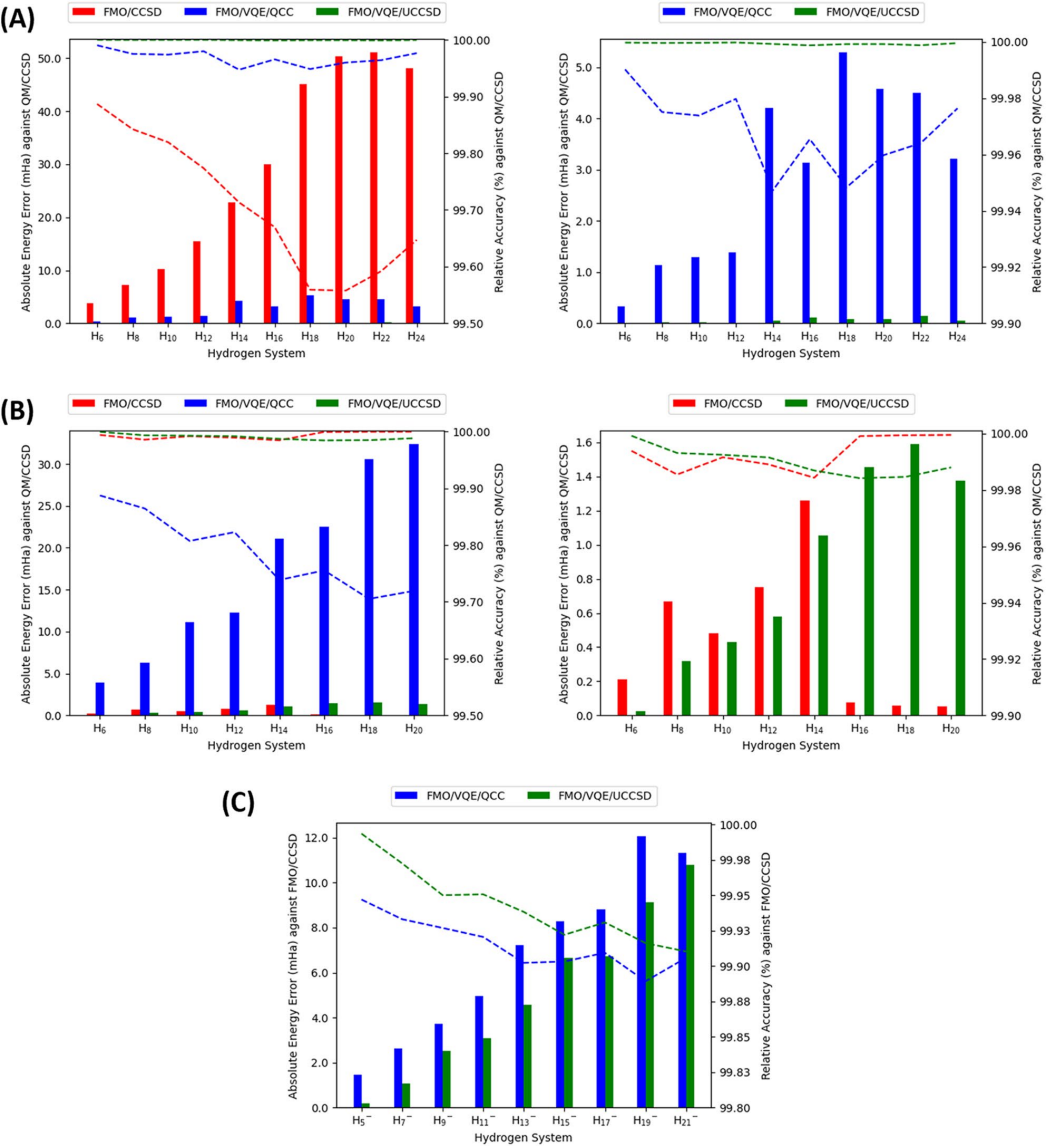
The Absolute Energy Errors and Relative Accuracy of FMO/VQE in Hydrogen Systems. (**A**) Results for neutral hydrogen systems using STO-3G basis set. This graph on the left side illustrates the absolute energy error (left axis) and relative accuracy (right axis), with QM/CCSD as the reference. A rescaled graph is provided on the right for enhanced visualization of the FMO/VQE/QCC and FMO/VQE/UCCSD methods. (**B**) Results for neutral hydrogen systems using 6-31G basis set. This graph on the left side also shows absolute energy errors and relative accuracy against QM/CCSD. The right graph has an adjusted y-axis scale for the FMO/CCSD and FMO/VQE/UCCSD. (**C**) Results for anionic hydrogen systems using 6-31G basis set. This graph uses FMO/CCSD as the reference, showing absolute energy errors and relative accuracy. Across all graphs, FMO/CCSD is shown in red, FMO/VQE/QCC in blue, and FMO/VQE/UCCSD in green. Absolute energy errors are represented by bar charts, while relative accuracy is shown with dotted line graphs.

The FMO/CCSD method is known to be a highly accurate fragment-based ab initio method and can provide a baseline for the accuracy assessment^42^. The FMO/CCSD with the STO-3G basis set showed relatively high absolute energy errors ranging from its lowest energy error (3.863 mHa) in ${{\text{H}}}_{6}$ to its highest energy error (51.012 mHa) in ${{\text{H}}}_{22}$ against the QM/CCSD, which is illustrated in Fig. 2A. The FMO/CCSD with the STO-3G basis set also achieved relative accuracies ranging from its lowest accuracy (99.55768%) in ${{\text{H}}}_{20}$ to its highest accuracy (99.88673%) in ${{\text{H}}}_{6}$. In contrast, the FMO/CCSD with the 6-31G basis set maintained low absolute energy errors and showed chemical accuracy through the various system sizes against the QM/CCSD in the neutral hydrogen clusters. The absolute energy errors range from its lowest energy error (0.051 mHa) in ${{\text{H}}}_{20}$ to its highest energy error (1.260 mHa) in ${{\text{H}}}_{14}$, which is illustrated in Fig. 2B. The relative accuracy of FMO/CCSD was the lowest (99.98437%) in ${{\text{H}}}_{14}$ and highest (99.99956%) in ${{\text{H}}}_{20}$. The combination of FMO/CCSD with the STO-3G basis set leads to relatively large absolute energy errors compared to the FMO/CCSD with the 6-31G basis set. This could be because the FMO/CCSD was not specifically designed for small basis sets such as STO-3G. The FMO/CCSD with the 6-31G basis set exhibited low absolute energy errors in the examination of the neutral hydrogen cluster systems. Despite the presence of a slight error, the FMO/CCSD approach with the 6-31G basis set remains a robust and effective method in evaluating the total absolute energies of neutral hydrogen cluster systems. Due to its demonstrated reliability, it can confidently be used as a point of reference, offering a viable alternative to the traditionally used QM/CCSD method.

Next, we compared the absolute energies from the FMO/VQE methods with two different ansätze (FMO/VQE/QCC and FMO/VQE/UCCSD) to those obtained using the QM/CCSD, which is illustrated in Fig. 2. The absolute energy errors of the FMO/VQE/QCC exhibited an increasing trend as the system size expanded. In the STO-3G basis set, the FMO/VQE/QCC showed the absolute energy errors from its lowest energy error of 0.332 mHa for ${{\text{H}}}_{6}$ to its highest energy error of 5.280 mHa for ${{\text{H}}}_{18}$ (Fig. 2A). Also, the FMO/VQE/QCC achieved relative accuracies ranging from its lowest value (99.94724%) in ${{\text{H}}}_{14}$ to its highest value (99.99025%) in ${{\text{H}}}_{6}$. This increase in error is even more striking when the 6-31G basis is employed (Fig. 2B). The absolute energy errors ranged from the lowest energy error (3.895 mHa) in ${{\text{H}}}_{6}$ to the highest energy error (35.160 mHa) in ${{\text{H}}}_{22}$, while the relative accuracies ranged from the lowest accuracy (99.70516%) in ${{\text{H}}}_{18}$ to the highest accuracy (99.88725%) in ${{\text{H}}}_{6}$. This suggests that the FMO/VQE/QCC ansatz, while effective in smaller systems, may struggle to maintain an equivalent level of accuracy as the complexity and size of the system grow.

In contrast, the FMO/VQE/UCCSD method demonstrates a noteworthy level of consistency and reliability across all system sizes, maintaining a remarkably low energy error even as the complexity of the hydrogen molecule cluster system increases. In the STO-3G basis set, the FMO/VQE/UCCSD showed absolute energy errors from its lowest energy error of 0.007 mHa for the ${{\text{H}}}_{6}$ to its highest energy error of 5.280 mHa for ${{\text{H}}}_{22}$. Furthermore, the FMO/VQE/UCCSD achieved relative accuracies from its lowest accuracy (99.99883%) in ${{\text{H}}}_{16}$ to its highest accuracy (99.99984%) in ${{\text{H}}}_{12}$. These relatively high accuracies and low errors in FMO/VQE/UCCSD are maintained even in the 6-31G basis set. The absolute energy errors ranged from the lowest energy error (0.026 mHa) in ${{\text{H}}}_{6}$ to the highest energy error (1.588 mHa) in ${{\text{H}}}_{18}$, while the relative accuracies ranged from the lowest accuracy (99.98420%) in ${{\text{H}}}_{16}$ to the highest accuracy (99.99925%) in ${{\text{H}}}_{6}$. This suggests a high degree of resilience and robustness in the FMO/VQE/UCCSD ansatz, making it an excellent choice for larger, more complex quantum systems, irrespective of basis sets. Its ability to handle larger and more complex quantum systems without a significant loss of accuracy highlights its potential applicability and effectiveness in the realm of quantum chemistry computations.

To compare the performance of the VQE and the FMO/VQE, the absolute energy error and relative accuracy for the neutral hydrogen cluster systems (${{\text{H}}}_{6}$, ${{\text{H}}}_{8}$, and ${{\text{H}}}_{10}$) were measured against QM/CCSD from the calculations with the STO-3G basis set, which is summarized in Table 3. Consistent with previous results, as the system size or the number of required qubits increased, the VQE/QCC method showed an increased error and reduced accuracy. In contrast, the FMO/VQE/QCC method not only reduced the required number of qubits compared to VQE/QCC, but it also decreased the absolute energy error to within the level of chemical accuracy and showed a relative accuracy of over 99.9%. On the other hand, the VQE/UCCSD method showed smaller errors than VQE/QCC, reaching the level of chemical accuracy, and also demonstrated an accuracy of over 99.99%. The FMO/VQE/UCCSD method, compared to VQE/UCCSD, reduced the required number of qubits, further minimized the absolute energy error, and reached a relative accuracy of over 99.999%. Therefore, we can conclude that the FMO/VQE method not only requires fewer qubits than the VQE methodology but also enhances its performance by decreasing the absolute energy error and increasing the relative accuracy.

**Table 3:** The error of VQE and FMO/VQE relative to QM/CCSD in neutral systems.

Metric	System	Basis set	VQE/QCC	FMO/VQE/QCC	VQE/UCCSD	FMO/VQE/UCCSD
Absolute energy error (mHa)	H_6_	STO-3G	1.781	0.332	0.001	0.007
	H_8_		3.558	1.132	0.052	0.016
	H_10_		7.233	1.286	0.133	0.016
Relative accuracy (%)	H_6_	STO-3G	99.94780	99.99025	100.00000	99.99980
	H_8_		99.92174	99.97511	99.99886	99.99965
	H_10_		99.87273	99.97386	99.99766	99.99972

Lastly, we applied the FMO/VQE method to an analysis of anionic hydrogen systems and compared the absolute energies from two FMO/VQE methods to those from the FMO/CCSD with a 6-31G basis set, which is summarized in Table 4 and illustrated in Fig. 2C. The absolute energy errors obtained from the FMO/VQE/QCC ranged from its lowest value of 1.461 mHa for the ${{\text{H}}}_{5}^{-}$ system to its highest value of 12.043 mHa for the ${{\text{H}}}_{19}^{-}$ system, while the relative accuracies of the FMO/VQE/QCC ranged from its lowest value of 99.88924% for the ${{\text{H}}}_{19}^{-}$ system to its highest value of 99.94698% for the ${{\text{H}}}_{5}^{-}$ system. On the other hand, the FMO/VQE/UCCSD yielded smaller absolute energy errors than the FMO/VQE/QCC, ranging from its lowest error (0.179 mHa) in ${{\text{H}}}_{5}^{-}$ to its highest error (13.181 mHa) in ${{\text{H}}}_{23}^{-}$. Furthermore, the FMO/VQE/UCCSD achieved even higher relative accuracies, from the lowest value of 99.90006% for ${{\text{H}}}_{23}^{-}$ to the highest value of 99.99350% for ${{\text{H}}}_{5}^{-}$. It demonstrates that both FMO/VQE/QCC and FMO/VQE/UCCSD consistently show good accuracy relative to the FMO/CCSD calculation for anionic systems. These methods exhibit reduced absolute energy errors and higher relative accuracies, indicating their effectiveness in approximating the reference FMO/CCSD calculation. These results highlight the potential of the FMO/VQE methods as reliable tools for quantum chemistry calculations in the context of anionic systems, offering accurate and efficient computational approaches in this domain.

**Table 4:** The error of FMO/VQE relative to FMO/CCSD in anionic systems.

System	Basis set	Absolute energy error (mHa)		Relative accuracy (%)	
		FMO/VQE/QCC	FMO/VQE/UCCSD	FMO/VQE/QCC	FMO/VQE/UCCSD
H_5_^−^	6-31G	1.461	0.179	99.94698	99.99350
H_7_^−^		2.622	1.075	99.93308	99.97256
H_9_^−^		3.713	2.536	99.92689	99.95006
H_11_^−^		4.958	3.080	99.92052	99.95064
H_13_^−^		7.234	4.570	99.90222	99.93823
H_15_^−^		8.283	6.666	99.90320	99.92209
H_17_^−^		8.805	6.723	99.90936	99.93079
H_19_^−^		12.043	9.114	99.88924	99.91618
H_21_^−^		11.335	10.796	99.90579	99.91027

## Discussion

The FMO method has been a critical development in managing large systems effectively for quantum chemistry calculations and significantly enhanced computational efficiency compared to conventional QM methods. It strategically dissects large systems into smaller, manageable fragments, maintaining reasonable accuracy throughout the process. Despite its advantages, the FMO method, particularly when coupled with the CC method and extensive basis sets, still requires a substantial computational cost, representing a substantial challenge for its broader applications. The incorporation of quantum computing into the FMO method presents a viable solution to overcome these limitations. In the present study, we have developed the FMO/VQE by merging the FMO framework with the VQE algorithm and demonstrated its usefulness with hydrogen cluster systems. The FMO/VQE can reduce the number of qubits required for the same molecular system, showcasing the feasibility of applying quantum computations to larger systems with fewer qubits.

Next, we focused on the validation of the FMO/VQE approach. The accuracy of the FMO/VQE was measured by applying it to hydrogen cluster systems, affirming its effectiveness. In general, the VQE tends to scale poorly for large molecules due to the need for repeated measurements or tomography to form the expected value of the Hamiltonian terms^51^. In contrast, the FMO/VQE approach determines the scope of the VQE application based on the size of the fragment, regardless of the system size. This aspect of FMO/VQE enables it to run VQE on larger systems with a smaller number of qubits, offering a significant advantage in handling more extensive molecular systems efficiently. However, based on our results, there is room for improvement and consideration. Firstly, in this study, the FMO/RHF was performed using a classical computer, and only the post-Hartree–Fock calculation, CCSD, was replaced by the VQE. The current implementation of FMO/VQE indeed relies on FMO/RHF results as the initial state. This dependency could lead to a bottleneck, even with the FMO/RHF approach. Therefore, it becomes essential to consider adapting the FMO/RHF component within a quantum computing framework, highlighting this as a crucial area for improvement in methodologies like FMO/VQE. By doing so, we can expect quantum acceleration, which would offer not only an opportunity to enhance computational efficiency but also further the fundamental understanding of quantum algorithm applications within complex molecular systems. Secondly, in the NISQ era, quantum hardware is inherently accompanied by significant quantum noise, which presents substantial challenges to the reliability and accuracy of quantum algorithms. Within this context, a critical aspect for the future development and evaluation of the FMO/VQE method is the exploration of its robustness in the presence of such quantum noise. This exploration should not be viewed merely as a technical necessity, but rather as a crucial step towards realizing the practical application of quantum computing in complex chemical systems. Hence, enhancing the stability of the FMO/VQE method in noisy environments would be a key future direction for its advancement. Thirdly, the FMO/VQE/QCC method underperformed compared to FMO/VQE/UCCSD in some cases. UCCSD is known for its accuracy and ability to capture a significant portion of electron correlation effects, while QCC, with its simpler structure, is more hardware-efficient but might offer a compromise in terms of accuracy. This balance between accuracy and resource efficiency may become particularly crucial in FMO, where compromising accuracy at the fragment level can have significant ramifications for the accuracy of overall system energy calculations. The choice of the VQE method should be tailored to the specific problem, taking into account both the accuracy requirements of the application and the hardware resources at hand. Furthermore, the exploration of enhancements in the FMO/VQE method opens new avenues for integrating additional quantum computing methods. While our focus has been on augmenting computational efficiency through VQE, there lies significant potential in exploring other quantum algorithms that could synergize with the FMO framework. This exploration is crucial, especially considering the rapidly evolving landscape of quantum computing and its applications in complex molecular systems. Given these considerations, the FMO/VQE would provide not only increased computational efficiency for the NISQ era but also offer a promising path for the practical application of quantum computers.

The QPE method is considered an algorithm to revolutionize the field of quantum chemistry and holds potential through exponential speedup. However, its effective deployment, specifically on fault-tolerant quantum computers, requires not only many qubits but also some challenges in harnessing practical quantum advantages. Firstly, the application of the QPE necessitates an accurate initial state for a molecular system, traditionally determined using classical computers. The process may be exponentially complex and important for larger molecular systems, such as biological systems because the computational cost of determining the correct ground state energy is contingent upon the overlap between the initial and target states. A decreasing overlap corresponds to a progressive increase in computational cost, thereby constituting a substantial impediment to practical application. Furthermore, even with an accurate initial state, the electronic structure problems in biological systems may not be strongly correlated, which could limit the expected quantum advantage from QPE alone. One potential strategy could involve fragmenting the total system into smaller subsystems to preserve the overall overlap, thereby facilitating a more effective application of the QPE. In this context, fragment-based quantum chemistry methods like the FMO approach may enhance the effectiveness of the QPE, by dividing the total system into smaller fragments to preserve the overall overlap, thereby complementing its strengths. Therefore, to fully leverage the practical benefits offered by quantum computing, it is important to develop new methodologies that can reduce computational costs, possibly through the compact representation of Hamiltonians.

Current quantum algorithms predominantly aim to achieve peak accuracy improvements, a characteristic not always necessary for industrial applications. For instance, the QPE will provide high accuracy in many quantum chemistry simulations through its quantum advantages, and one may want to utilize the advantages to accelerate the drug discovery process. However, many drugs, typically small and closed-shell organic molecules, often lack strong correlations. Thus, while the theoretical pursuit of maximal accuracy is commendable, it may not be the primary requirement for practical applications such as drug design. From a more applied perspective, using perturbation theory-level results coupled with a multitude of single-point calculations can more efficiently predict thermodynamic quantities such as binding affinity. Looking further, if we could directly compute thermodynamic properties such as free energy using a thermal ensemble through quantum computers, it could be a game-changing strategy in industrial applications. Current quantum algorithms may offer extreme accuracy, but in the world of practical application, other factors such as computational efficiency and direct computation of industrially relevant properties can often supersede the necessity for accuracy. In conclusion, while the peak accuracy provided by quantum algorithms is essential in certain contexts, practical industrial applications often require a balance between accuracy and efficiency. Therefore, future research and development in quantum computing should also consider these requirements to make quantum computing a truly game-changing technology in the industry.

## Conclusion

Our newly developed algorithm, FMO/VQE, demonstrates remarkable proficiency in conducting quantum chemistry simulations within the constraints of current quantum computing technologies, effectively surpassing the limitations of standard VQE methods. Specifically, we applied the FMO/VQE to several hydrogen systems and validated its accuracy and efficiency, where the FMO/VQE showed the capability to efficiently utilize a limited number of qubits, making it exceptionally well-suited for larger molecular systems. In contrast to traditional VQE methods, our FMO/VQE's scalability is significantly enhanced by its ability to apply VQE within a defined scope based on fragment size, independent of the overall system size. This attribute allows FMO/VQE to tackle larger systems with fewer qubits. Furthermore, our FMO/VQE not only showcases impressive efficiency in handling larger systems but also demonstrates accuracy that is on par with traditional VQE. As such, our findings herald a promising path for the future of quantum chemical simulations in the age of quantum computing, potentially accommodating the analysis of increasingly complex systems as quantum computing technologies continue to advance and the availability of qubits grows.

## Data Availability

All results in this study are available in the GitHub repository (https://github.com/QuNovaComputing/OpenFMO-VQE).
